# Discitis, Diabetes, and Endocarditis: A Challenging Clinical Triad

**DOI:** 10.7759/cureus.83702

**Published:** 2025-05-08

**Authors:** Nandana N Hegde, Hariharan G, Mohd Asif, Sravan Kumar Gaddamedi, Yughandar S, Sharada V Kutty

**Affiliations:** 1 Department of General Medicine, All India Institute of Medical Sciences, Mangalagiri, Mangalagiri, IND; 2 Department of Cardiology, All India Institute of Medical Sciences, Mangalagiri, Mangalagiri, IND; 3 Department of Radiology, All India Institute of Medical Sciences, Mangalagiri, Mangalagiri, IND

**Keywords:** aortic valve vegetation, brucellosis, clinical, infective endocarditis, native valve disease, occupational exposure, pyrexia of unknown origin (puo), spondylodiscitis, tuberculosis, zoonosis

## Abstract

A 58-year-old male farmer from southern India with a history of diabetes mellitus presented with a one-year history of fever and progressive lower back pain. He was initiated on antitubercular therapy (ATT) for suspected tuberculous spondylitis. Despite three months of ATT, his symptoms worsened, with progressive constitutional symptoms and features of heart failure. Further evaluation revealed aortic valve vegetation and persistent lumbar spondylodiscitis. Serological and imaging findings, along with occupational exposure to livestock, led to a diagnosis of brucellosis with infective endocarditis and spondylodiscitis. The patient’s condition deteriorated despite supportive care and antibiotics as per treatment guidelines. This case highlights the diagnostic challenges of brucellosis in endemic regions and underscores the importance of considering zoonotic infections in patients with atypical presentations of spondylodiscitis and endocarditis, in regions where both tuberculosis and brucellosis are endemic.

## Introduction

Brucellosis is a zoonotic infection caused by *Brucella* species. It is transmitted primarily through direct contact with infected animals or the consumption of unpasteurized dairy products. The disease remains endemic in regions with close human-animal interaction, including regions such as parts of Asia, the Mediterranean, and the Middle East [1]. Brucellosis often presents with nonspecific symptoms such as fever, night sweats, fatigue, and musculoskeletal pain. It can affect nearly any organ system, including the osteoarticular, cardiovascular, and genitourinary systems, leading to clinical overlap with other chronic infections such as tuberculosis (TB) [2].

TB, particularly spinal TB (Pott’s disease), often presents with chronic back pain, low-grade fever, weight loss, and night sweats. Brucellosis can similarly involve the spine, resulting in spondylodiscitis and clinical features that closely resemble those of Pott’s disease. This overlap makes distinguishing between the two conditions particularly challenging in TB-endemic regions. TB spondylitis typically demonstrates contiguous vertebral involvement with intervertebral disc narrowing, paravertebral abscesses, and kyphotic deformity, whereas brucellar spondylodiscitis more commonly affects the lumbar spine and may exhibit less aggressive bone destruction and preserved disc spaces in early stages [3]. Laboratory differentiation can be aided by blood cultures, standard agglutination tests (SAT) for brucellosis, and histopathological or molecular tests for *Mycobacterium tuberculosis*.

Complications such as spondylodiscitis and infective endocarditis in brucellosis can lead to significant morbidity and mortality if not promptly diagnosed and treated [3].

This case report describes a patient with brucellosis who presented with both spondylodiscitis and infective endocarditis. He was initially managed as a case of tuberculous spondylitis. The case underscores the diagnostic challenge in differentiating these two infections in endemic areas and highlights the need for a high index of suspicion for brucellosis in select patients.

## Case presentation

A 58-year-old male farmer from southern India with a three-year history of diabetes mellitus presented to the emergency department with fever, progressive lower back pain, and shortness of breath. His symptoms had persisted for over a year, with intermittent low-grade fever and insidious onset of back pain that worsened over time. The pain, initially rated 2/10, progressed to 6/10, radiating to the posterior thigh and leg (Wong Baker FACES pain rating scale). It was accompanied by a sensation of numbness and tingling in the same region. There were no features of bowel or bladder dysfunction. He reported that the pain impeded his activities of daily living and disturbed his sleep.

After eight to nine months of the aforementioned symptoms, the patient initially reported to another healthcare facility for evaluation. The patient underwent a comprehensive fever workup, which included blood cultures, urine cultures, chest X-ray, urine analysis, erythrocyte sedimentation rate (ESR), C-reactive protein (CRP), procalcitonin, and tropical infections. These investigations remained inconclusive. An MRI of the dorsolumbar spine was performed, which revealed L4-L5 spondylodiscitis. The patient was started on the four-drug antitubercular therapy (ATT) for suspected tuberculous spondylitis on a clinicoradiological basis.

Despite three months of adherence to ATT, his symptoms worsened, with persistent fever and chills, weight loss of approximately eight kilograms, and progressive dyspnea advancing to New York Heart Association (NYHA) class IV symptoms. He noted that shortness of breath had rapidly progressed over a period of three weeks and was associated with streaky hemoptysis and left-sided precordial discomfort. One week prior to the presentation at our center, the patient noted bilateral lower limb swelling that progressively worsened to generalized edema. 

On admission, the patient was febrile, pale, and had bilateral pitting pedal edema. His pulse rate was 96 bpm, regular, large volume with a collapsing nature. He was normotensive and tachypneic. Elevated jugular venous pulsations were observed 4 cm above the sternal angle. The patient was alert and responsive. There were bilateral crepitations and wheezing on auscultation. A grade II/IV, high-pitched, soft-blowing early diastolic decrescendo murmur was noted at the neoaortic space (left third intercostal space), best heard in a sitting and leaning forward position, in full expiration, and with the diaphragm of the stethoscope.

There was significant tenderness over the L4-L5 region, with a positive straight leg raising test (SLRT) at 50°. Clinical examination was suggestive of aortic regurgitation with left ventricular failure. There were no other peripheral signs of aortic regurgitation or infective endocarditis. There were no focal neurological deficits. 

The laboratory investigations revealed the following. The patient had normocytic normochromic anemia (8.7 g/dL) and thrombocytopenia (117.8 x 10³/μL). Renal function tests revealed elevated blood urea nitrogen (25.75 mg/dL), blood urea (55.1 mg/dL), serum creatinine (2.5 mg/dL), and serum uric acid levels (14.6 mg/dL). Urine routine and microscopy revealed mild proteinuria and hematuria. A renal ultrasonography was performed, which revealed normally sized kidneys without any structural abnormalities. The N-terminal pro-B-type natriuretic peptide (NT-proBNP) levels were significantly elevated at 30,235.3 pg/mL, suggesting heart failure. Elevated CRP (40.7 mg/dL), D-dimer (3.8 mcg/mL), and increased ESR levels (30 mm in one hr) were noted (Table 1).

**Table 1:** Laboratory investigations at the time of the presentation. NT-proBNP: N-terminal pro-B-type natriuretic peptide; ESR: erythrocyte sedimentation rate; CRP C-reactive protein; RDW: red cell distribution width

Investigation	Value	Normal range
Hemoglobin	8.70 g/dL	13-17 g/dL
Hematocrit	28.55%	40-50%
RDW	18.51%	11.6-14%
Platelet count	117.8 *10^3^/ μL	150-410 *10^3^/ μL
Urine blood	3+	Negative
Urine protein	2+	Negative
Urine pus cells	4-5 /Hpf	0-4 /Hpf
Urine RBC	8-9 /Hpf	0-3 / Hpf
Blood urea nitrogen	25.75 mg/dL	6-20 mg/dL
Blood urea	55.1 mg/dL	12.84-42.8 mg/dL
Serum creatinine	2.5 mg/dL	0.62-1.1 mg/dL
Serum uric acid	14.6 mg/dL	4.4- 7.6 mg/dL
Serum potassium	2.8 mEq/L	3.5 - 5.1 mEq/L
NT-proBNP	30235.3 pg/mL	<125 pg/mL
CRP	40.7 mg/dL	0.3 - 8.6 mg/dL
D-dimer	3.8 mcg/mL	<0.5 mcg/mL
ESR	30 mm in 1 hr	<13 mm in 1 hr

Three paired blood samples were inoculated in aerobic and anaerobic media using the automated BACTEC systems (Becton, Dickinson and Company (BD), New Jersey, USA). These remained sterile at day five of incubation. A tuberculin skin test with purified protein derivative was performed and was negative.

**Figure 1:**
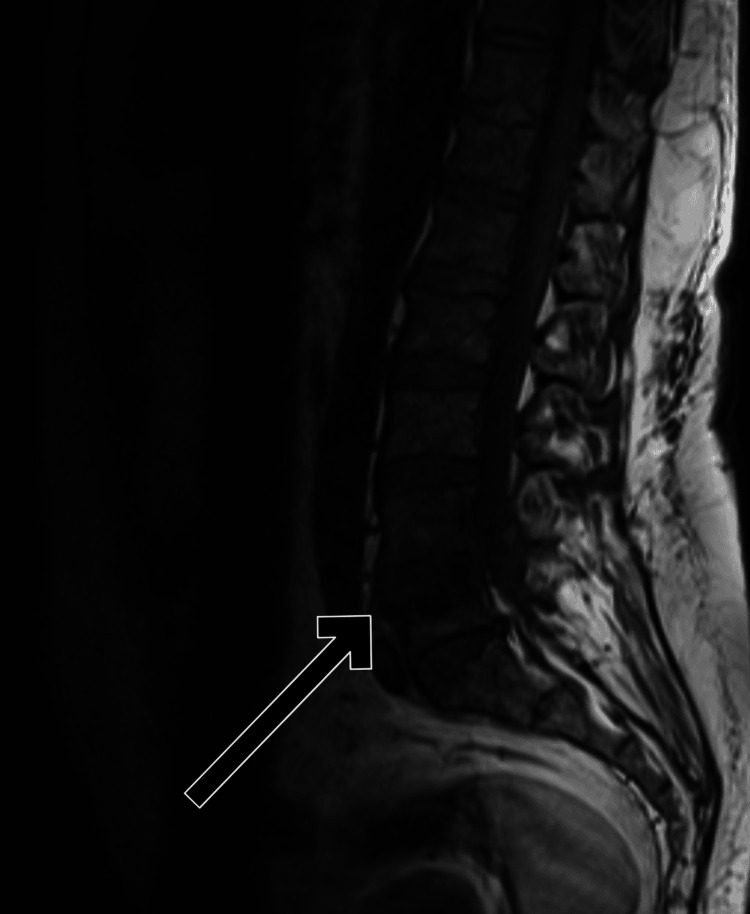
Sagittal T1 image demonstrating destruction of the L4-5 intervertebral disc space with T1 hypointense fluid within the disc. There is T1 hypointense edema at the opposing end plates of the L4 and L5 vertebral bodies (arrow).

**Figure 2:**
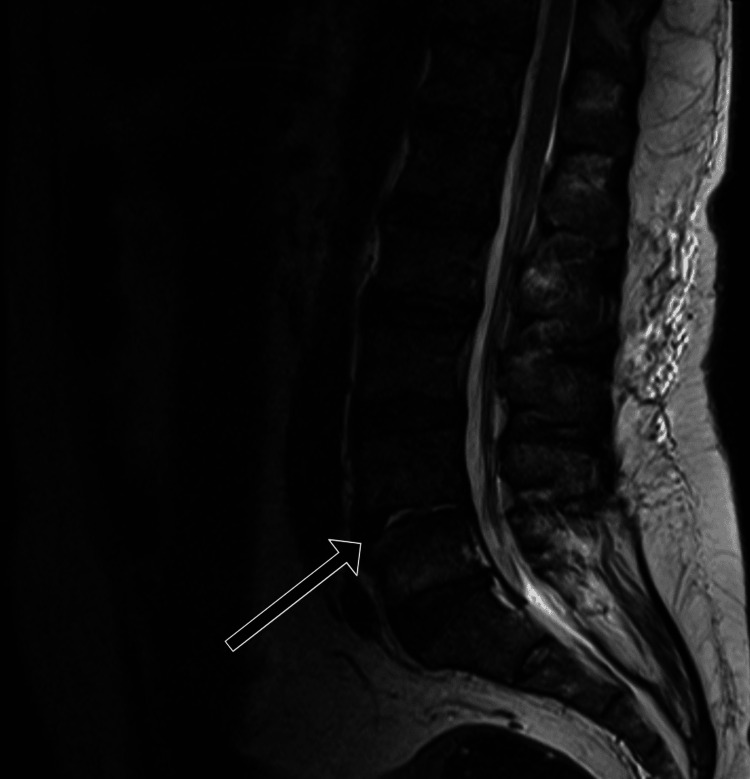
Sagittal T2 image demonstrating destruction of the L4-5 intervertebral disc space with T2 hyperintense fluid within the disc. There is T2 hyperintense edema at the opposing end plates of the L4 and L5 vertebral bodies (arrow).

**Figure 3:**
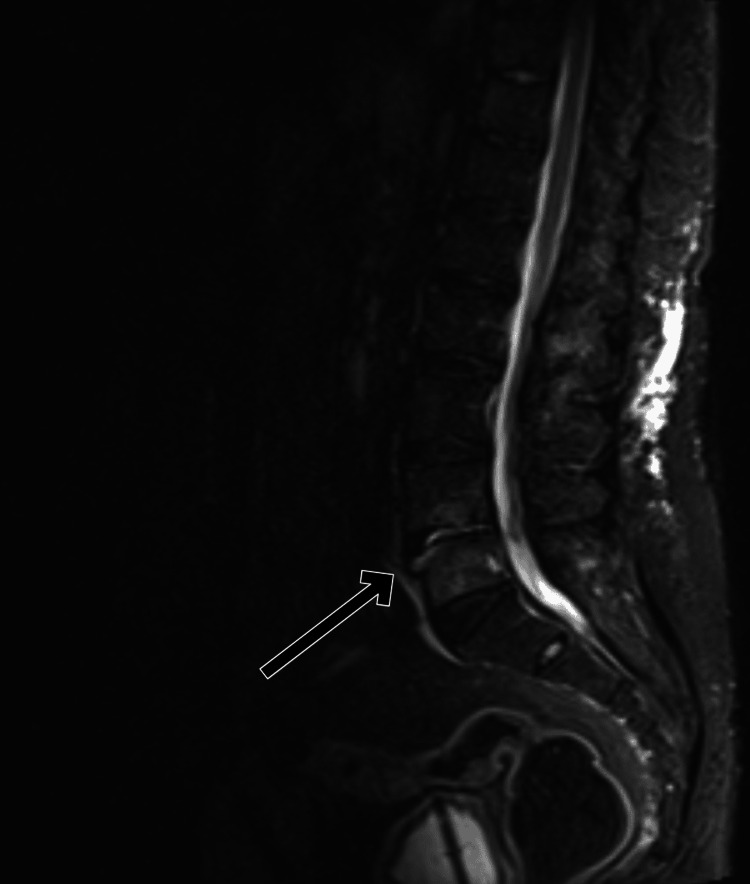
Sagittal STIR image showing hyperintense fluid within the L4–L5 intervertebral disc, with destruction of the opposing end plates, which demonstrated hyperintense edema (arrow). STIR: short tau inversion recovery

MRI of the spine shows collapse of the L4-L5 intervertebral disc with fluid collection, indicating residual spondylodiscitis and lumbar spondylosis. There was destruction of the disc space and edema at the L4 and L5 vertebral end plates, with T1 hypointensity and T2 hyperintensity on imaging, consistent with inflammation and disc degeneration (Figures 1-3).

**Figure 4:**
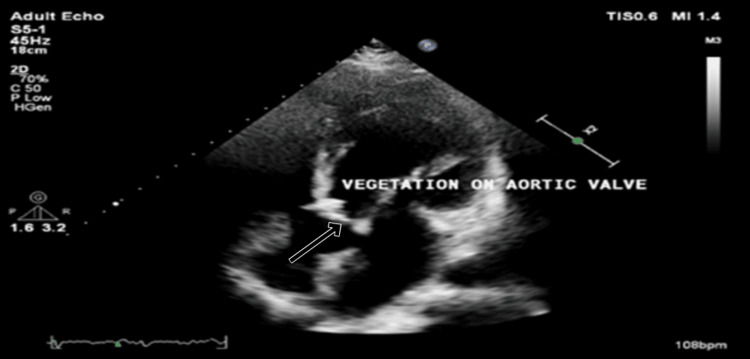
Apical 5-chamber (A5C) view on 2D echocardiography demonstrating vegetation on the aortic valve (arrow).

Due to the progressive shortness of breath, a 2D transthoracic echo was performed, which revealed a large vegetation of size 13*9 mm on the aortic valve with moderate to severe aortic regurgitation (Figure 4).

**Figure 5:**
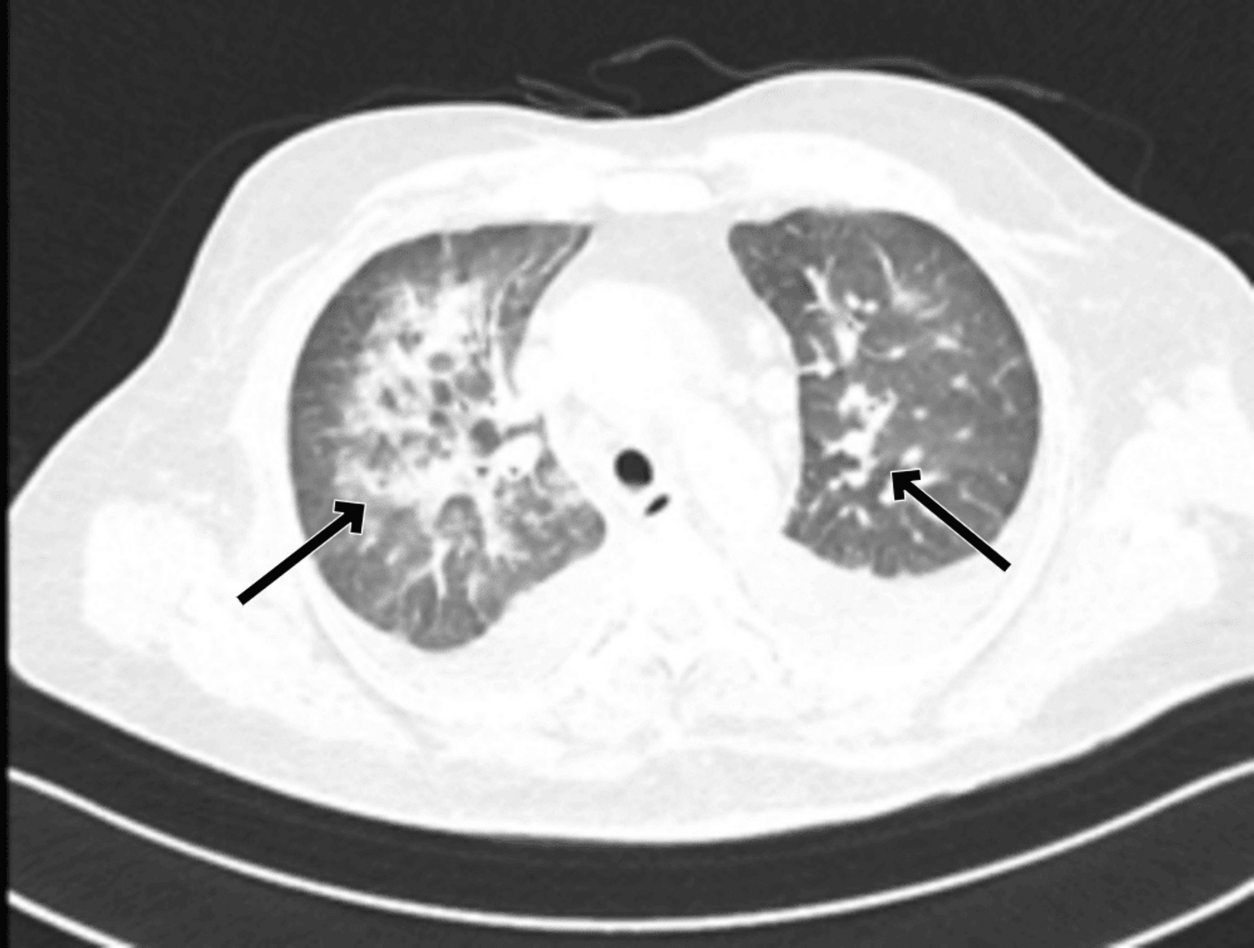
Axial HRCT section of the chest at the level of the bilateral upper lobes, demonstrating confluent ground-glass opacities and consolidation in perihilar and central locations with relative sparing of the lung periphery (arrows). HRCT: high-resolution computed tomography

**Figure 6:**
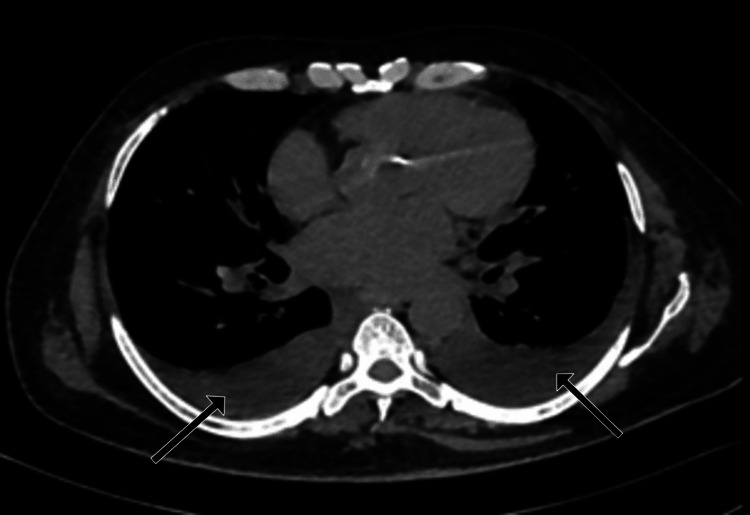
Axial section of the chest in the mediastinal window at the level of the bilateral lower lobes, demonstrating bilateral moderate pleural effusion (arrows).

High-resolution computed tomography (HRCT) of the chest revealed confluent areas of ground glass opacities with perihilar distribution and bilateral moderate pleural effusion suggestive of cardiogenic pulmonary edema (Figures 5, 6).

Given the patient’s occupational exposure to livestock and failure to respond to ATT, brucellosis was suspected. Serological tests confirmed the diagnosis (Table 2). *Brucella* IgM was positive .

**Table 2:** Brucella serology report. ELISA: enzyme-linked immunoassay

Test description	Results	Units	Biological reference ranges
*Brucella* IgM Antibody (ELISA method)	26.09	NTU	<9.0 : Negative; 9.0 to 11.0: Equivocal; >11.0: Positive

The diagnosis of infective endocarditis in this patient was based on the modified Duke criteria. The major criterion was evidence of endocardial involvement, as echocardiography revealed aortic valve vegetation.

The minor criteria included a predisposing condition-occupational exposure to livestock (significant in this case); fever ≥38°C, documented during multiple clinical visits; vascular phenomena, which were not observed; immunological phenomena, which were not observed; and microbiological evidence, with positive serological testing for *Brucella *(meeting the microbiological support criterion in the context of culture-negative endocarditis).

Based on the modified Duke criteria, the presence of one major and at least two minor criteria supports a possible infective endocarditis diagnosis, consistent with *Brucella* endocarditis presentations reported in endemic regions.

The blood cultures remained sterile at day 21 of incubation, which is not uncommon in brucellosis due to its fastidious nature and prior empirical antibiotic exposure. As a result, antibiotic susceptibility testing could not be conducted. A biopsy from the bony lesion and tissue culture were considered; however, the procedure was not performed as the patient was clinically unstable at that time. Given his deteriorating condition, invasive diagnostic interventions were deferred. The diagnosis in this patient was confirmed through positive serological testing (SAT), supported by clinical and radiological findings. 

Despite supportive care and appropriate antibiotics (rifampicin, streptomycin, doxycycline), the patient’s condition continued to deteriorate. He developed intractable heart failure and was deemed unfit for surgical intervention due to the advanced nature of his illness. Unfortunately, he succumbed to the disease one week later.

## Discussion

Brucellosis is a zoonotic infection caused primarily by the genus *Brucella*, which is transmitted to humans from infected animals, typically through direct contact with animal tissues or the consumption of unpasteurized dairy products. The disease is endemic in regions with high rates of animal husbandry, particularly in the Mediterranean, the Middle East, and parts of Asia and Latin America [4].

The clinical presentation of brucellosis is diverse and often nonspecific, making diagnosis challenging. The most common symptoms include fever, fatigue, sweating, malaise, myalgias, and arthralgias, which can resemble a variety of other infectious or inflammatory diseases. In its acute form, brucellosis may present with undulant fever (intermittent fever spikes), which is often characteristic of the disease. However, chronic brucellosis can lead to complications, including osteoarticular involvement (e.g., spondylodiscitis), hepatosplenomegaly, and endocarditis [5]. It remains a significant public health challenge in areas where people work closely with livestock, such as farmers, veterinarians, and abattoir workers. This case highlights the importance of considering brucellosis in patients with spondylodiscitis and infective endocarditis, especially those with occupational exposure to livestock [6].

While there is data regarding the association of type 2 diabetes mellitus (T2DM) and TB, the same cannot be said for brucellosis. It is known that individuals with T2DM may exhibit compromised neutrophil functions and an increased susceptibility to infections. The intricate relationship between T2DM and the immune system significantly impacts disease progression and patient outcomes. In addition to being a metabolic disorder, T2DM is a condition that causes chronic inflammation and immune dysfunction [7]. 

Since our patient was diabetic, we hypothesize that a prolonged course and poorer outcomes would be expected. This could be attributed to chronic immune dysregulation as discussed.

Brucellosis is diagnosed primarily through a combination of serological tests, such as the rose Bengal test (RBT), complement fixation test (CFT), or enzyme-linked immunosorbent assay (ELISA), and cultures. In some cases, molecular diagnostic methods, such as polymerase chain reaction (PCR), may be used for confirmation [8].

The sensitivity of RBT is reported to be 80-95%, and the specificity is usually between 90-98% in endemic regions, although false positives can occur due to cross-reactivity with other zoonotic infections such as Q fever or leptospirosis [9]. The sensitivity of IgG ELISA has been reported to be 85-95%, and the specificity ranges between 90-100%, making it one of the most widely used serological methods. IgM ELISA can show increased sensitivity in acute infections, although false positives are possible. CFT has a sensitivity of 75-95%, depending on the stage of infection, and a specificity of approximately 80-95%. However, it is less widely used due to its complexity and need for specialized lab settings. The sensitivity of *Brucella* PCR varies from 70% to 95% depending on the sample type (e.g., blood, tissue, or bone marrow) and the PCR technique used [10]. PCR tends to have greater sensitivity when samples are collected in the acute phase of infection and from more invasive specimens such as bone marrow or lymph nodes. The specificity of *Brucella* PCR tests is typically 90% to 100%. PCR is generally very specific, as it detects *Brucella* DNA directly and avoids cross-reactivity with other pathogens. However, false positives may arise in areas with a high prevalence of *Brucella*-like organisms, which can lead to some cross-reactivity. The culture method is often considered the gold standard for the diagnosis of brucellosis. The sensitivity generally ranges from 50% to 85%, depending on the sample type and the stage of infection. Blood cultures are commonly used, but their sensitivity may be lower in chronic cases [11]. Culture has a specificity close to 100%, making it a definitive diagnostic method when *Brucella* is isolated. However, culture is time-consuming, with results taking up to one to three weeks, and may be negative in patients who have been treated with antibiotics prior to testing (Table 3) [12].

**Table 3:** Comparison of diagnostic tests for brucellosis (sensitivity, specificity, and clinical utility). ELISA: enzyme-linked immunosorbent assay; PCR: polymerase chain reaction

Test	Sensitivity	Specificity	Clinical utility
Rose Bengal test	80 to 95%	90 to 98%	Rapid screening; useful in field/outbreak settings
Standard agglutination test	95 to 100%	95 to 100%	Commonly used; titres ≥1:160 often considered significant
ELISA (IgM, IgG)	85 to 95%	90 to 100%	Differentiates acute (IgM) from chronic (IgG); quantitative and more specific
PCR	70 to 95%	90 to 100%	Useful in early or chronic disease; detects *Brucella* DNA directly
Complement fixation test	75 to 95%	80 to 95%	Confirms active infection; complement-dependent
Blood cultures	50 to 85%	100%	Definitive diagnosis; slow-growing; biosafety level 3 pathogen

Imaging studies such as MRI or CT scans are useful in diagnosing complications (particularly musculoskeletal involvement) such as spondylodiscitis or osteomyelitis. MRI is a highly sensitive imaging technique and is the choice of modality to rule out spondylodiscitis in cases where there is high clinical suspicion.

Multiple spectra of findings can be seen in brucellar spondylodiscitis (BSD). Laiyong Tu et al. studied 72 cases of BSD and identified that lumbosacral vertebrae were the most common site of involvement, and endplate osteolysis was seen in 75% of cases [13]. Vertebral body osteogenesis and bony bridge formations were also identified. B. Zulkif et al. studied 22 patients with spondylodiscitis and reported that the majority involved a single vertebra. Abscess formation was observed in three patients, and posterior longitudinal ligament elevation was observed in four patients [6]. A Chinese multicenter study included 615 cases [14], in which intervertebral disc space involvement was observed in the majority of cases. Paravertebral and epidural abscess formation was also observed in approximately half of these patients.

Hui Guo et al. compared 26 cases of BSD and a similar number of tubercular spondylodiscitis cases and noted a significantly lower incidence of vertebral body destruction, posterior vertebral body cortex deformities, abscess, and dead bone formation in patients with BSD [15]. F. Hammami et al. studied 73 patients with tubercular spondylodiscitis and 44 patients with BSD cases and reported similar findings [16]. Liu X et al. reported that preserved vertebral height and new bone formation at the endplates are more common in patients with BSD [17]. Similar findings were also noted by Xu Q. [18]. Imaging differentials include tubercular spondylodiscitis, fungal spondylodiscitis, pyogenic spondylodiscitis, and vertebral metastasis.

Infective endocarditis is a rare but severe complication of brucellosis, occurring in 1-2% of cases [15]. The presence of large vegetations on the aortic valve, as observed in our patient, is associated with high mortality, particularly in the absence of surgical intervention [16]. 

The treatment of brucellosis typically involves a combination of antibiotics. The standard regimens include doxycycline and rifampin, which are used for a prolonged period to ensure eradication of the organism. In severe cases, or in patients with complications such as endocarditis or spondylodiscitis, the therapy may be extended or supplemented with additional antibiotics such as streptomycin or gentamicin. Surgical intervention may be required for certain complications, such as endocarditis or abscess formation.

The complications of brucellosis can be severe, particularly in immunocompromised individuals or those who do not receive timely treatment. Infective endocarditis, osteomyelitis, and neurological manifestations (such as meningitis) are among the most serious sequelae. Chronic brucellosis, if left untreated or inadequately treated, can result in long-term disability, especially due to joint and spine involvement [19].

Despite effective treatment options, brucellosis can be challenging to diagnose because of its wide range of symptoms and overlap with other infectious diseases. In areas where TB is endemic, such as many regions of Asia, brucellosis may be mistaken for TB, as both diseases can present with chronic fever, weight loss, and musculoskeletal involvement.

The patient’s initial misdiagnosis of tuberculous spondylitis highlights the limitations of empirical ATT in endemic regions. *Brucella* spondylodiscitis typically presents with chronic back pain, fever, and weight loss, often mimicking TB [13]. However, key differentiating features include a history of animal contact, slower progression, and less severe vertebral destruction than TB [6]. Imaging findings such as preserved vertebral height and new bone formation are more suggestive of brucellosis [14].

This case highlights the need for a high index of suspicion for brucellosis in patients with atypical presentations of spondylodiscitis and endocarditis, particularly in endemic regions. Early diagnosis and appropriate treatment are crucial to prevent complications and improve outcomes [20].

## Conclusions

This case highlights the diagnostic challenges in differentiating brucellosis from TB, particularly in endemic regions where both infections can present with similar clinical manifestations, such as fever, musculoskeletal pain, and spondylodiscitis. While TB remains a more common cause of spinal infection, brucellosis should be considered in patients with relevant occupational exposure, such as those working with livestock. Key distinguishing features include the absence of classic TB signs (e.g., cavitary lung lesions or typical granulomatous changes on histology) and the presence of aortic valve vegetations in brucellosis, which may not be as common in TB. Serological testing for *Brucella* and imaging play pivotal roles in confirming the diagnosis. This case underscores the importance of maintaining a broad differential diagnosis and highlights the need for clinicians to be vigilant in recognizing zoonotic infections, particularly in individuals with risk factors for exposure. We should further explore the pathophysiological interplay between T2DM and *Brucella* infection to optimize evidence-based risk stratification and management strategies for mitigating comorbid disease progression in affected populations.
